# Circulating Ghrelin, Nesfatin-1, and Vaspin Levels Across Glycemic Control Categories in Type 2 Diabetes Mellitus: A Cross-Sectional Study

**DOI:** 10.3390/biomedicines14092055

**Published:** 2026-09-12

**Authors:** Suheda Akkus, Orkun Saricam, Murat Kavruk, Gulcin Turkmen Sariyildiz, Fatma Busra Benguboz, Berna Yilmaz Sirin, Elif Esra Altuner, Ali Dogan Dursun, Veli Cengiz Ozalp

**Affiliations:** 1 Department of Kinesiology, Institute of Health Sciences, Atilim University, Ankara 06830, Türkiye; akkusuheda@outlook.com (S.A.); ali.dursun@atilim.edu.tr (A.D.D.); 2 Department of Internal Medicine, Ankara Pursaklar State Hospital, Ankara 06145, Türkiye; orkunsar@hotmail.com; 3 Institute of Nanotechnology and Biotechnology, Istanbul University-Cerrahpasa, Istanbul 34500, Türkiye; murat.kavruk@iuc.edu.tr; 4 Health Biotechnology Joint Research and Applications Center of Excellence, Istanbul 34220, Türkiye; 5 Department of General Surgery, Medicana International Ankara Hospital, Ankara 06510, Türkiye; gsariyildiz@medicana.com.tr; 6 Vocational School of Health Services, Atilim University, Ankara 06830, Türkiyeberna.yilmazsirin@atilim.edu.tr (B.Y.S.); 7 Program of Medical Laboratory Techniques, Department of Medical Services and Technology, Vocational School of European, University of Kocaeli Health and Technology, Kocaeli 41275, Türkiye; elif.altuner@kocaelisaglik.edu.tr; 8 Centre for Laboratory Animal Breeding and Experimental Research (ATÜDEM), Atilim University, Ankara 06830, Türkiye; 9 Home Care Services, Medicana International Ankara Hospital, Ankara 06510, Türkiye; 10 Department of Biomedical Engineering, Atlas University, Istanbul 34408, Türkiye

**Keywords:** diabetes mellitus, adipokine, ghrelin, nesfatin-1, vaspin, HbA1c, glycemic control, biomarker

## Abstract

**Background/Objectives:** Diabetes mellitus is characterized by chronic hyperglycemia, insulin resistance, and low-grade inflammation, and adipose-tissue-derived adipokines are increasingly implicated as regulators of glucose homeostasis. This study compared circulating ghrelin, nesfatin-1, and vaspin levels across three glycemic-control categories defined by HbA1c and evaluated their association with glycemic status. **Methods:** In this cross-sectional comparative study, serum ghrelin, nesfatin-1, and vaspin were measured by enzyme-linked immunosorbent assay (ELISA) in 161 adults stratified into three groups: HbA1c 6.0–8.4% (*n* = 55), HbA1c ≥ 8.5% (n = 52), and non-diabetic controls with HbA1c < 6.0% (n = 54). Distributional assumptions were tested with the Kolmogorov–Smirnov and Shapiro–Wilk tests. Because all three adipokines deviated from normality, the primary omnibus comparison was the Kruskal–Wallis test, followed by Dunn’s post hoc test with Bonferroni adjustment; effect sizes are reported with 95% confidence intervals. Group differences were additionally examined after adjustment for age and sex, and HbA1c was analysed as a continuous variable. **Results:** Ghrelin (Kruskal–Wallis *p* = 0.726) and vaspin (*p* = 0.364) did not differ among the three groups. Nesfatin-1 differed significantly (H = 15.16, *p* < 0.001, ε^2^ = 0.095) and non-monotonically: concentrations were higher in the HbA1c 6.0–8.4% group than in controls (median 824 versus 650 pg/mL; Dunn *p* = 0.018) and than in the HbA1c ≥ 8.5% group (824 versus 600 pg/mL; *p* < 0.001), whereas the HbA1c ≥ 8.5% and control groups did not differ (*p* = 0.909). The nesfatin-1 difference persisted after adjustment for age and sex (*p* < 0.001) and after excluding controls with impaired fasting glucose (*p* < 0.001). Within the diabetic participants, nesfatin-1 correlated inversely with continuous HbA1c (ρ = −0.373, *p* < 0.001). **Conclusions:** Nesfatin-1, but not ghrelin or vaspin, differed significantly between HbA1c-defined glycemic-control categories in a non-monotonic manner, with the highest concentrations in the intermediate-HbA1c group. Because body mass index, diabetes duration, and antidiabetic medication were not available, and because the design is cross-sectional, these between-group differences cannot be interpreted as a within-person trajectory or attributed to glycemic status independently of adiposity. These exploratory findings require confirmation in larger, prospective, BMI- and medication-adjusted cohorts before nesfatin-1 can be considered a candidate biomarker.

## 1. Introduction

Diabetes mellitus (DM) is a chronic metabolic disorder characterized by persistent hyperglycemia arising from defective insulin secretion, insulin action, or both [1]. Global prevalence has risen sharply: an estimated 537 million adults were living with diabetes in 2021, a figure projected to reach 783 million by 2045 [2]. Type 2 diabetes (T2DM), the predominant form in adults, is driven by insulin resistance compounded by progressive β-cell dysfunction, with obesity and sedentary lifestyle as major modifiable risk factors [3]. Beyond glycemic dysregulation, T2DM is increasingly understood as a disease intertwined with chronic low-grade inflammation, oxidative stress, and altered adipose-tissue endocrine signaling [4].

Adipose tissue is an active endocrine organ that secretes a range of bioactive peptides collectively termed adipokines, which regulate energy homeostasis, insulin sensitivity, and inflammatory tone [4]. Because several adipokines are altered early in the course of insulin resistance, they have been proposed both as candidate biomarkers of glycemic deterioration and as potential therapeutic targets [5]. However, findings across the adipokine literature are frequently inconsistent, and the cellular mechanisms linking specific adipokines to glycemic control remain incompletely defined. In order to tackle this paucity of information, this study focuses on three adipokines with plausible, but still debated, roles in glucose homeostasis: ghrelin, nesfatin-1, and vaspin.

Ghrelin, first isolated from rat gastric extracts as the endogenous ligand of the growth hormone secretagogue receptor [6], is produced predominantly by the gastric fundus and, to a lesser extent, by the pancreas, intestine, and other tissues [7]. Beyond its orexigenic role, ghrelin regulates insulin secretion, hepatic glucose output, and energy balance, and disrupted ghrelin signaling has been implicated in obesity and insulin resistance [8]. Experimental work indicates that ghrelin suppresses glucose-stimulated insulin release [9], and some population-based studies report that low circulating ghrelin is independently associated with insulin resistance and T2DM [10], while others report reduced ghrelin specifically in obese individuals with T2DM relative to obese non-diabetic and healthy comparators [11]. More recent work has framed these observations within the ghrelin–GHSR–LEAP2 axis: obesity and T2DM are characterised by reduced acylated ghrelin and increased LEAP2, particularly in the presence of insulin resistance [12,13], and a meta-analysis of 34 studies (1863 participants) confirmed that both acylated and des-acylated ghrelin are lower in obesity than in healthy controls [14]. Notably, the only quantitative synthesis addressing ghrelin and insulin resistance directly found a significant inverse correlation only in normal-fasting-glucose subgroups, with no correlation once fasting glucose was elevated [15]—a subgroup effect of direct relevance to an HbA1c-stratified design such as ours. The clinical significance of these associations in humans remains undetermined [16,17].

Nesfatin-1 is an 82-amino-acid peptide derived from the precursor protein nucleobindin-2, first described in 2006, with anorexigenic and putative antihyperglycemic properties [18]. It is widely expressed in the central nervous system and peripheral tissues and participates in appetite regulation, lipid metabolism, and glucose homeostasis [19,20]. Nesfatin-1 and ghrelin originate from the same gastric X/A-like cells and exert largely opposing effects on energy and glucose metabolism, which is one rationale for measuring them in the same cohort [21]. Clinical studies have produced conflicting results: some report lower circulating nesfatin-1 in individuals with established T2DM relative to healthy controls, while others report elevated nesfatin-1 specifically in newly diagnosed, treatment-naïve patients, with levels declining after initiation of antidiabetic therapy—a pattern attributed to a compensatory response that is expressed early but attenuates as disease and treatment progress [22,23]. Case–control series have reported significantly reduced nesfatin-1 in established T2DM with intermediate values in prediabetes [24,25], and the most recent meta-analysis (8 studies; 305 patients and 205 controls) confirmed a significant association between serum nesfatin-1 and T2DM but with very high between-study heterogeneity (I^2^ = 98%) [26], indicating that the direction and magnitude of the association remain unresolved.

Vaspin (visceral adipose tissue-derived serpin, SERPINA12) is a more recently identified adipokine with insulin-sensitizing properties, first isolated from an obese, insulin-resistant rat model [27]. Proposed mechanisms include activation of the IRS/PI3K/Akt/GLUT signaling axis and inhibition of the IκBα/NF-κB inflammatory pathway [28]. Elevated vaspin has been reported in obesity, polycystic ovary syndrome, and early insulin resistance, consistent with a compensatory role, whereas some cohort and meta-analytic data suggest declining vaspin with longer diabetes duration and more severe metabolic derangement [29,30]. Recent large-scale evidence has strengthened, rather than resolved, the picture: in 10,052 PURE and 7840 ORIGIN participants, higher circulating vaspin was associated with a 16% greater risk of incident T2DM per standard-deviation increase, and Mendelian randomisation linked elevated vaspin to reduced gluteofemoral adiposity [31]; a separate genome-wide and Mendelian randomisation study (n = 7446) indicated that vaspin causally raises triglycerides and LDL cholesterol [32]. Vaspin is likewise elevated in obesity [33] and in gestational diabetes (22 studies; SMD 0.72, 95% CI 0.44–1.00) [34]. Importantly, no meta-analysis of circulating vaspin concentrations in type 2 diabetes has been published since 2014 [30]. As with ghrelin and nesfatin-1, findings across studies are not fully concordant, and human data stratified specifically by glycemic severity remain limited.

Given this heterogeneous and at times contradictory evidence base, we conducted a cross-sectional comparative study of circulating ghrelin, nesfatin-1, and vaspin concentrations among individuals with type 2 diabetes and intermediate glycemic control (HbA1c 6.0–8.4%), individuals with type 2 diabetes and poor glycemic control (HbA1c ≥ 8.5%), and non-diabetic controls (HbA1c < 6.0%), with the aim of clarifying whether, and how, these three adipokines differ across categories of glycemic severity in this population.

## 2. Materials and Methods

### 2.1. Study Design and Participants

This was a single-center, cross-sectional comparative study conducted at Ankara Pursaklar State Hospital (Ankara, Türkiye). We use the term cross-sectional throughout: all measurements were obtained at a single time point, and participants were classified by strata of a continuous exposure variable (HbA1c) rather than being sampled on the basis of a disease outcome and matched to controls, which is what a case–control design would require. A total of 161 adult participants were enrolled and stratified into three groups by glycated hemoglobin (HbA1c): Group A, type 2 diabetes with intermediate glycemic control (HbA1c 6.0–8.4%, n = 55); Group B, type 2 diabetes with poor glycemic control (HbA1c ≥ 8.5%, n = 52); and a non-diabetic control group (HbA1c < 6.0%, n = 54).

Rationale for the HbA1c strata: All participants in Groups A and B had a previously established, physician-documented diagnosis of type 2 diabetes recorded in the hospital information system; no participant was newly diagnosed at the time of sampling. The lower bound of Group A (HbA1c 6.0%) corresponds to the upper limit applied to the control group, so that the three strata are mutually exclusive and exhaustive. The 8.5% cut-point separating Groups A and B was selected pragmatically as a threshold above which glycemia is unambiguously and substantially above the general adult target of HbA1c < 7% recommended by the American Diabetes Association, and by approximately 1.5% or more—the margin at which guidelines advise initial combination rather than stepwise pharmacotherapy [35,36]. We emphasise that 8.5% is not itself a guideline-defined diagnostic or treatment threshold, and that Group A spans a range of glycemic control (HbA1c 6.0–8.4%) that is clinically heterogeneous; the group is therefore described as having intermediate rather than adequate or good control, and HbA1c is additionally analysed as a continuous variable (Section 2.4) precisely because categorisation discards information.

Controls were adults attending the same institution with HbA1c < 6.0% and no documented history of diabetes mellitus, and none were receiving glucose-lowering therapy. Participants with a known diagnosis of diabetes, pregnancy, or active malignancy were not enrolled in the control group. We note explicitly, as a limitation rather than a design feature, that fasting plasma glucose was not applied as an additional inclusion criterion for controls, and that we did not systematically exclude controls on the basis of obesity, cardiovascular disease, chronic kidney disease, inflammatory disorders, or medications affecting metabolism. The consequences of this are quantified in Section 3.2 and Section 3.5 and discussed in Section 4.5.

Exclusion criteria applied to all groups were age < 18 years, pregnancy or lactation, known type 1 diabetes or other specific types of diabetes, active malignancy, and end-stage renal disease requiring dialysis.

Across the three groups, the sex distribution was closely comparable (71–75% female; χ^2^ = 0.182, *p* = 0.913), whereas the control group was substantially younger than either diabetic group (Table 1). Body mass index (BMI) was not recorded in the source dataset, and no surrogate measure of adiposity—waist circumference, weight, or self-reported body weight—was available for retrospective retrieval. Because all three adipokines examined here are strongly adiposity-dependent, this is the principal limitation of this study and constrains the interpretation of every between-group comparison reported below. Diabetes duration and current antidiabetic medication were likewise unavailable. The age difference between groups is addressed analytically by adjustment (Section 2.4).

### 2.2. Ethical Approval

This study was conducted in accordance with the Declaration of Helsinki and approved by the Institutional Review Board of Atılım University (approval number: 604.01.02-439; date: 30 April 2025). Written informed consent was obtained from all participants prior to enrolment.

### 2.3. Biochemical Analyses

Venous blood samples were collected after an overnight fast of at least 8 h, centrifuged, and serum was stored at −80 °C until analysis. Serum ghrelin, nesfatin-1, and vaspin concentrations were determined by ELISA using commercial kits from Elabscience Biotechnology (Houston, TX, USA), each analyte with its own dedicated kit and its own standard curve: human ghrelin (catalogue E-EL-H1919; competitive ELISA; standard curve 0.156–10 ng/mL; sensitivity 0.09 ng/mL), human nesfatin-1 (E-EL-H2373; sandwich ELISA; standard curve 15.63–1000 pg/mL; sensitivity 9.38 pg/mL), and human vaspin (E-EL-H1762; sandwich ELISA; standard curve 62.5–4000 pg/mL; sensitivity 37.5 pg/mL). Manufacturer-reported precision was intra-assay CV 4.3–6.8% and inter-assay CV 7.5–9.0% for ghrelin, intra-assay CV 4.6–6.8% and inter-assay CV 6.1–7.7% for nesfatin-1, and intra-assay CV 4.2–6.1% and inter-assay CV 7.1–8.6% for vaspin; the manufacturer states a repeatability coefficient of variation below 10% for all three kits. Standards and serum samples were incubated in the supplied antibody-precoated 96-well microplates (37 °C, 90 min), followed by sequential incubation with biotinylated detection antibody (37 °C, 60 min) and horseradish-peroxidase–streptavidin conjugate (37 °C, 30 min), with washing steps between stages. Color development was achieved with TMB substrate (15–30 min, 37 °C) and stopped with an acidic stop solution; optical density was read at 450 nm on a microplate reader (BioTek, Winooski, VT, USA). Samples were assayed in duplicate and concentrations were interpolated from the analyte-specific standard curve; the vaspin assay required a 1:250 serum predilution to bring readings within the working range of the curve, and the reported vaspin concentrations incorporate this dilution factor, whereas ghrelin and nesfatin-1 were assayed without predilution. All samples for a given analyte were run on the same plate lot and, wherever possible, within the same assay run, with the three study groups distributed across plates to avoid confounding of group with batch.

### 2.4. Statistical Analysis

Data were analyzed using SPSS version 29 (IBM Corp., Armonk, NY, USA) with confirmatory computation in Python 3 (SciPy 1.17, statsmodels 0.15). The distribution of each continuous variable was assessed within each group using both the Kolmogorov–Smirnov (Lilliefors) and the Shapiro–Wilk test, together with inspection of skewness, kurtosis, and histograms; a variable was treated as non-normally distributed if normality was rejected in at least one group by the Shapiro–Wilk test. On this basis ghrelin, nesfatin-1, vaspin, HbA1c, fasting glucose, eGFR, triglycerides, HDL-C, LDL-C, ALT, and AST were treated as non-normally distributed, and age and total cholesterol as normally distributed (per-variable results are given in Table S1). Homogeneity of variance was assessed with Levene’s test.

The analysis follows a conventional omnibus-then-post hoc framework. For each variable a single three-group omnibus test was performed first: the Kruskal–Wallis test for non-normally distributed variables, one-way ANOVA for normally distributed variables with homogeneous variances, and Welch’s ANOVA where Levene’s test indicated unequal variances. For the three adipokines this rule selected the Kruskal–Wallis test; the corresponding parametric tests (one-way and Welch’s ANOVA) were nonetheless computed for all analytes and are reported alongside as a sensitivity analysis, irrespective of Levene’s result. Pairwise post hoc comparisons were performed only if the omnibus test was significant, using Dunn’s test with Bonferroni adjustment after Kruskal–Wallis, Tukey’s HSD after ANOVA, and the Games–Howell procedure after Welch’s ANOVA (Table S3). Because each family of three pairwise comparisons is adjusted within its own omnibus test, a global Bonferroni correction across all nine adipokine comparisons is not required, and α = 0.05 is used throughout; the unadjusted pairwise results are given in Table S2.

Descriptive statistics are reported as mean ± standard deviation (SD) and as median with interquartile range (IQR); for non-normally distributed variables the median (IQR) is the primary summary. Effect sizes are reported for every comparison: ε^2^ (H/(N − 1)) for Kruskal–Wallis and η^2^ for ANOVA as omnibus effect sizes, and, for pairwise comparisons, the rank-biserial correlation together with the Hodges–Lehmann median difference and its distribution-free 95% confidence interval for rank-based tests, and Hedges’ g and the difference in means, each with a 95% confidence interval, for parametric tests. Confidence intervals for median differences were additionally verified by bias-corrected bootstrapping with 10,000 resamples.

Three sets of supporting analyses were added. First, because HbA1c is intrinsically continuous, Spearman rank correlations between each adipokine and HbA1c were computed in the whole cohort and within the diabetic participants (Groups A and B combined), and the Jonckheere–Terpstra test was used to test for a monotonic ordered trend across control → Group A → Group B. Second, multivariable linear regression modelled each adipokine on HbA1c, age, and sex, with a quadratic HbA1c term added and tested by a nested F test to assess non-monotonicity; analysis of covariance (ANCOVA) estimated the group effect adjusted for age and sex, and was repeated on van der Waerden normal scores as a rank-based, distribution-free equivalent. Third, prespecified sensitivity analyses repeated the primary comparisons (i) after excluding control participants with fasting plasma glucose ≥ 100 mg/dL, (ii) after excluding values lying more than three interquartile ranges below the first or above the third quartile of their group (Tukey’s extreme-outlier criterion), (iii) after natural-log transformation of vaspin, and (iv) stratified by sex. Sex distribution was compared with the chi-square test. Two-sided *p*-values are reported; because no formal a priori power calculation was performed, all findings are interpreted as exploratory.

## 3. Results

### 3.1. Baseline Characteristics

Demographic characteristics of the three study groups are summarized in Table 1. The sex distribution did not differ among groups (74.5%, 71.2%, and 74.1% female in Group A, Group B, and controls, respectively; χ^2^ = 0.182, df = 2, *p* = 0.913). Age, by contrast, differed markedly (one-way ANOVA F(2,158) = 30.27, *p* < 0.001, η^2^ = 0.277): controls were on average 13.3 years younger than Group A (95% CI 8.0–18.6, Tukey *p* < 0.001) and 16.7 years younger than Group B (95% CI 11.3–22.1, *p* < 0.001), whereas the two diabetic groups did not differ from one another (*p* = 0.289). This age imbalance is carried forward into all adjusted analyses (Section 3.6).

### 3.2. Clinical and Biochemical Parameters

Clinical and biochemical characteristics, with formal between-group inferential testing, are given in Table 2. As expected, HbA1c and fasting glucose increased progressively from controls through Group A to Group B, with all three pairwise contrasts significant for both variables (Dunn *p* < 0.001 throughout). Triglycerides differed among groups (Kruskal–Wallis *p* = 0.001), being higher in both diabetic groups than in controls (Group A versus control *p* = 0.015; Group B versus control *p* = 0.001) but not differing between the diabetic groups (*p* = 1.000). In contrast, total cholesterol (*p* = 0.394), HDL-C (*p* = 0.269), LDL-C (*p* = 0.113), ALT (*p* = 0.670), and AST (*p* = 0.659) did not differ significantly among the groups; the numerically higher LDL-C and transaminase values in Group B therefore do not constitute evidence of a group difference.

eGFR differed among groups (Kruskal–Wallis *p* < 0.001) and was, unexpectedly, lowest in Group A (median 84, IQR 71–96 mL/min/1.73 m^2^), intermediate in Group B (88, 81–97), and highest in controls (98, 88–105); post hoc, both diabetic groups differed from controls (Group A *p* < 0.001; Group B *p* = 0.014) but not from each other (*p* = 0.592). This ordering is almost entirely attributable to age: age correlated strongly and inversely with eGFR across the cohort (ρ = −0.671, *p* < 0.001), and in an ANCOVA of eGFR on group, age, and sex the age term dominated (F = 111.0, *p* < 0.001) while the adjusted difference between Group A and controls disappeared (β = −1.9 mL/min/1.73 m^2^, 95% CI −7.4 to 3.6, *p* = 0.498). After age adjustment, Group B in fact had a slightly higher eGFR than controls (β = +6.1, 95% CI 0.3–11.9, *p* = 0.041), a pattern consistent with glomerular hyperfiltration in poorly controlled diabetes; three participants in Group B had an eGFR ≥ 120 mL/min/1.73 m^2^, and moderately reduced renal function (eGFR <60) was confined to the diabetic groups (Group A n = 5, Group B n = 4, controls n = 0). The unadjusted eGFR ordering therefore reflects the age imbalance between groups rather than an inverse relationship between renal function and glycemic burden.

Fasting plasma glucose in the control group deserves separate comment. Although the control group was defined by HbA1c < 6.0% and had a mean fasting glucose of 100.4 ± 11.9 mg/dL (median 99, IQR 92–108), 23 of 54 controls (42.6%) had fasting glucose in the impaired fasting glucose range of 100–125 mg/dL and 2 (3.7%) had a value ≥ 126 mg/dL; 17 of 54 (31.5%) had an HbA1c of 5.7–5.9%. Only 22 of 54 controls (40.7%) met neither the fasting glucose nor the HbA1c criterion for prediabetes. The control group is therefore best characterised as non-diabetic rather than as strictly euglycemic. A prespecified sensitivity analysis restricted to the 29 controls with fasting glucose < 100 mg/dL is reported in Section 3.5, and the implications are discussed in Section 4.5.

### 3.3. Adipokine Concentrations and Between-Group Comparisons

**Figure 1:**
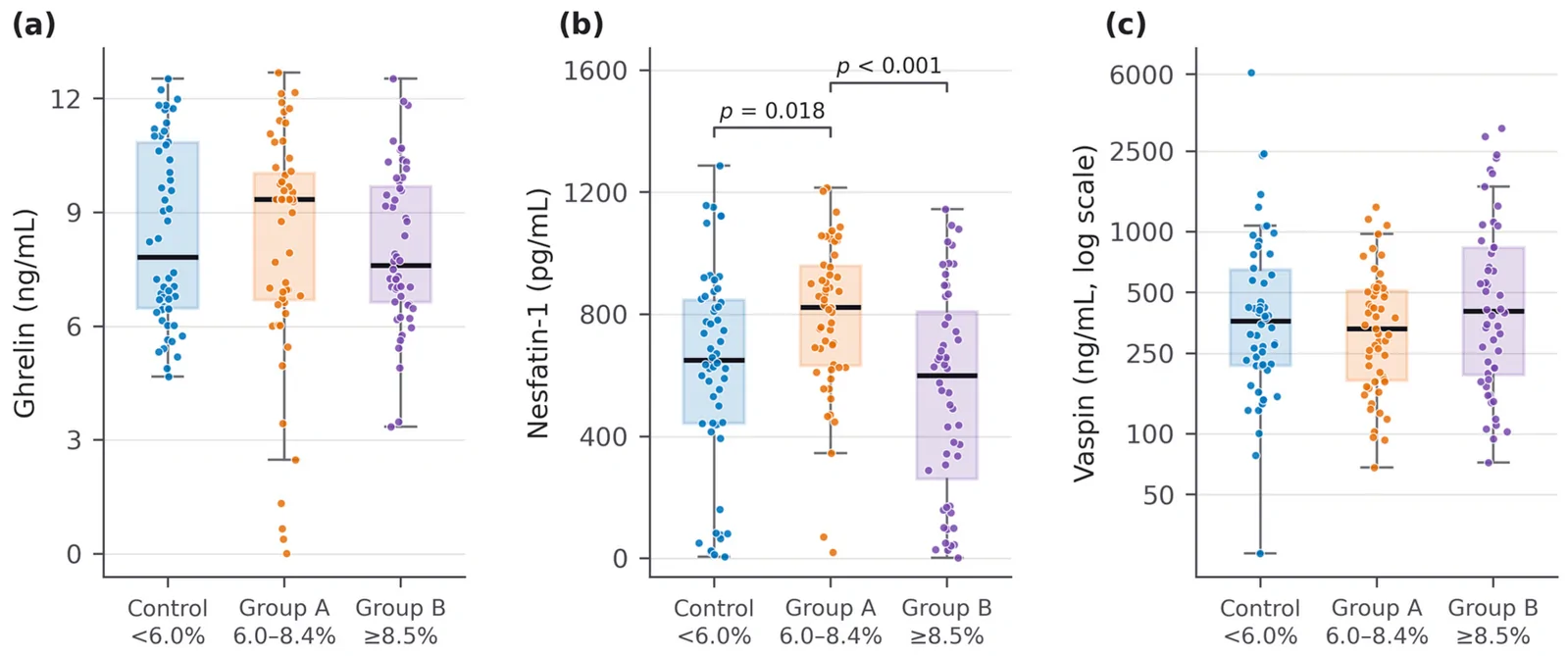
Distribution of circulating (**a**) ghrelin, (**b**) nesfatin-1, and (**c**) vaspin across the three HbA1c-defined groups. Boxes show the median and interquartile range, whiskers extend to 1.5 × IQR, and individual participant values are overlaid. *p*-values are Dunn’s post hoc test with Bonferroni adjustment, shown only where the Kruskal–Wallis omnibus test was significant. The vaspin panel uses a logarithmic ordinate because of the marked right-skew of that analyte.

Adipokine concentrations by group are shown in Table 3 and in Figure 1; omnibus tests and post hoc comparisons with effect sizes are given in Table 4. All three adipokines departed from normality in at least one group (Shapiro–Wilk *p* ≤ 0.023; Table S1), and the dispersion of vaspin was extreme, with coefficients of variation of 70%, 109%, and 148% in Group A, Group B, and controls, respectively. The Kruskal–Wallis test was therefore the primary omnibus test for all three analytes.

Ghrelin did not differ among the three groups (H(2) = 0.641, *p* = 0.726, ε^2^ = 0.004), and no post hoc testing was warranted. Effect sizes for all three pairwise contrasts were negligible (rank-biserial |r| ≤ 0.10; Hodges–Lehmann median differences of 0.04, −0.22, and 0.41 ng/mL, all with confidence intervals spanning zero; Table 4). One value in Group A (0.016 ng/mL) fell below the assay’s stated limit of detection; excluding it changed neither the descriptive statistics materially (Group A mean 8.32 ± 2.90 ng/mL) nor the omnibus result (*p* = 0.634).

Nesfatin-1 differed significantly among the three glycemic categories (H(2) = 15.16, *p* < 0.001, ε^2^ = 0.095) in a non-monotonic pattern. Concentrations were highest in Group A (median 824.2 pg/mL, IQR 631.7–959.4), intermediate in controls (650.1, 443.0–847.5), and lowest in Group B (599.7, 259.9–808.1). Post hoc, Group A exceeded controls (Dunn z = 2.745, adjusted *p* = 0.018; Hodges–Lehmann difference 155.2 pg/mL, 95% CI 45.2–265.8; rank-biserial r = 0.307) and Group B (z = 3.753, adjusted *p* < 0.001; Hodges–Lehmann difference 247.7 pg/mL, 95% CI 120.8–380.5; r = 0.418), whereas Group B and controls did not differ (z = −1.030, adjusted *p* = 0.909; 95% CI for the median difference −224.2 to 52.0). Expressed parametrically, the same contrasts correspond to Hedges’ g of 0.57 (95% CI 0.19–0.95) for Group A versus controls and 0.83 (0.43–1.22) for Group A versus Group B—a moderate and a large effect, respectively—and to −0.23 (−0.61 to 0.15) for Group B versus controls. Bootstrap confidence intervals for the median differences (10 000 resamples) excluded zero for both significant contrasts and included zero for Group B versus controls. Consistent with a non-monotonic rather than a graded relationship (Table S4), the Jonckheere–Terpstra test for an ordered trend across control → Group A → Group B was not significant (z = −0.956, *p* = 0.339).

Vaspin did not differ among the three groups (H(2) = 2.019, *p* = 0.364, ε^2^ = 0.013), and all pairwise effect sizes were small with confidence intervals spanning zero (rank-biserial |r| ≤ 0.17; Table 4). Group B had the numerically highest arithmetic mean (693.9 ± 755.5 ng/mL) but the group medians were closely similar (Group A 331.1, Group B 404.5, controls 360.8 ng/mL), and the discrepancy between the mean and median ordering reflects a marked right-skew rather than a group difference: the standard deviation exceeded the mean in both Group B and controls, and five values across the cohort met Tukey’s extreme-outlier criterion (more than three interquartile ranges above the third quartile of their group), the largest being 6145 ng/mL in a control participant. Because a mean-based test is not robust to this degree of skew, a parametric omnibus test applied to the untransformed data would have yielded a nominally significant result (Welch’s ANOVA *p* = 0.015) that is an artefact of these few extreme values; three prespecified sensitivity analyses confirm the null finding. After removal of the five extreme values, the Kruskal–Wallis *p*-value was 0.517. After natural-log transformation, which rendered all three distributions normal (Shapiro–Wilk *p* ≥ 0.309), the geometric means were 318.8, 428.6, and 376.1 ng/mL and one-way ANOVA on the log scale was not significant (F = 1.52, *p* = 0.221), with a Group B to Group A geometric-mean ratio of 1.34 (95% CI 0.96–1.88). Vaspin also showed no correlation with continuous HbA1c (Section 3.6). We therefore conclude that vaspin did not differ across glycemic categories in this cohort, and that the dominant feature of the vaspin data is inter-individual heterogeneity rather than any glycemia-related signal.

### 3.4. Sex and Age as Determinants of Adipokine Concentrations

Because the cohort was predominantly female (73.3% overall) and sex is a recognised determinant of adipokine concentrations, sex effects were examined explicitly. Ghrelin was higher in women than in men (median 9.1 versus 7.0 ng/mL, *p* = 0.008), and the difference for nesfatin-1 was substantial (799.5 versus 393.6 pg/mL, *p* < 0.001); vaspin did not differ by sex (383.6 versus 347.2 ng/mL, *p* = 0.143). In the ANCOVA models (Table 5) sex was the strongest single predictor of nesfatin-1 (F = 56.1, *p* < 0.001; adjusted difference for men −359.6 pg/mL, 95% CI −454.4 to −264.8), exceeding the group effect in magnitude. Age, in contrast, was not independently associated with any of the three adipokines (all *p* ≥ 0.275). The dominance of sex over glycemic category as a determinant of nesfatin-1 is an incidental but noteworthy observation of this study, and it is the reason the group comparisons are reported both unadjusted and adjusted.

### 3.5. Sensitivity Analyses

The nesfatin-1 finding was robust to every sensitivity analysis performed. Restricting the control group to the 29 participants with fasting plasma glucose < 100 mg/dL left the omnibus result unchanged (Kruskal–Wallis *p* < 0.001) and the Group A versus control contrast significant (Mann–Whitney *p* = 0.030), with a near-identical control median (658.6 versus 650.1 pg/mL in the full control group); the ghrelin and vaspin null findings likewise persisted (*p* = 0.529 and *p* = 0.372). Stratifying by sex, the group difference in nesfatin-1 was present and of similar shape in women (H = 10.65, *p* = 0.005; medians 888, 661, and 758 pg/mL in Group A, Group B, and controls) and in men (H = 11.39, *p* = 0.003; medians 632, 150, and 404 pg/mL), indicating that it is not driven by the sex imbalance. In a female-only ANCOVA adjusted for age (n = 118) the group effect remained significant (F = 5.04, *p* = 0.008). A rank-based ANCOVA on van der Waerden normal scores, which makes no distributional assumption, gave the same result (group F = 8.49, *p* < 0.001 for nesfatin-1; *p* = 0.814 for ghrelin and *p* = 0.257 for vaspin).

### 3.6. Continuous and Adjusted Analyses

Treating HbA1c as a continuous variable (Table 5), no adipokine correlated with HbA1c across the whole cohort (ghrelin ρ = 0.007, *p* = 0.925; nesfatin-1 ρ = −0.084, *p* = 0.290; vaspin ρ = 0.067, *p* = 0.401), which is itself consistent with a non-monotonic relationship in which controls occupy an intermediate position. Within the diabetic participants alone (Groups A and B, n = 107), however, nesfatin-1 was inversely and significantly correlated with HbA1c (ρ = −0.373, *p* < 0.001), whereas ghrelin (ρ = −0.044, *p* = 0.654) and vaspin (ρ = 0.164, *p* = 0.092) were not. In multivariable regression on HbA1c, age, and sex, the linear HbA1c coefficient for nesfatin-1 was −37.2 pg/mL per 1% HbA1c (95% CI −58.7 to −15.7, *p* < 0.001; model R^2^ = 0.300), and adding a quadratic HbA1c term produced a negative coefficient of borderline significance (β = −8.32, nested F(1,156) = 3.46, *p* = 0.065; R^2^ = 0.315)—directionally consistent with the inverted-U pattern seen in the group analysis, but not established by this test alone. In ANCOVA adjusted for age and sex, the group effect on nesfatin-1 remained highly significant (F = 10.96, *p* < 0.001), with Group A exceeding controls by 141.7 pg/mL (95% CI 29.3–254.1, *p* = 0.014) and Group B not differing significantly from controls (−100.4 pg/mL, 95% CI −219.8 to 19.0, *p* = 0.099). The adjusted group effect was non-significant for ghrelin (F = 0.19, *p* = 0.829) and for vaspin (F = 2.60, *p* = 0.077).

## 4. Discussion

### 4.1. Ghrelin

Ghrelin concentrations were statistically indistinguishable across glycemic categories in this cohort (medians 9.35, 7.61, and 7.82 ng/mL in Group A, Group B, and controls; Kruskal–Wallis *p* = 0.726), and all pairwise effect sizes were negligible with confidence intervals comfortably spanning zero; so, this is an informative null rather than an underpowered one. The finding contrasts with population-based reports linking low circulating ghrelin to insulin resistance and T2DM [10] and with experimental evidence that ghrelin suppresses glucose-stimulated insulin secretion [9], and with recent reviews describing reduced acylated ghrelin and increased LEAP2 in T2DM [12,13]. It is, however, consistent with the broader observation that ghrelin’s metabolic associations in humans are less robust and more heterogeneous than in animal models [8,16], and it aligns specifically with the meta-analytic finding that the inverse ghrelin–insulin resistance correlation is detectable in normal-fasting-glucose subgroups but disappears once fasting glucose is elevated [15]—precisely the population sampled here, in which two of three groups are hyperglycemic by definition. Two unmeasured factors constrain this interpretation and are more consequential than the null result itself. Adiposity correlates inversely with ghrelin independently of glycemic status [11,14], and BMI was not available in this dataset; ghrelin is also modified by antidiabetic therapy, including insulin and GLP-1 receptor agonist exposure, which was likewise unrecorded. Total rather than acylated ghrelin was measured, and the acylated fraction carries most of the receptor-mediated metabolic activity, which may further dilute any true signal. Our data therefore support the narrower conclusion that total ghrelin does not distinguish HbA1c-defined glycemic categories in this population, rather than the stronger claim that ghrelin is unrelated to diabetes.

### 4.2. Nesfatin-1

Nesfatin-1 was the only one of the three adipokines to differ significantly between glycemic-control categories, and it did so non-monotonically: concentrations were higher in the intermediate-HbA1c group than in either the poorly controlled group or the non-diabetic controls, which did not differ from one another. The pattern was robust to the choice of statistical framework (rank-based and parametric), to adjustment for age and sex, to sex stratification, to restriction of the control group to participants with normal fasting glucose, and to bootstrap resampling, and it was corroborated within the diabetic participants by an inverse correlation with continuous HbA1c (ρ = −0.373, *p* < 0.001). The absence of a monotonic ordered trend (Jonckheere–Terpstra *p* = 0.339) together with a borderline negative quadratic HbA1c term (*p* = 0.065) points in the same direction: whatever relationship exists between nesfatin-1 and glycemia in this cohort is not a straight line.

This configuration is compatible with reports of elevated nesfatin-1 in newly diagnosed or less advanced T2DM, attributed to a compensatory anorexigenic and insulin-sensitising response, alongside evidence of lower nesfatin-1 with longer disease duration and antidiabetic treatment exposure [22,23], and with case–control series in which prediabetic values lie between those of controls and established T2DM [24,25]. The heterogeneity of this literature is substantial—the most recent meta-analysis reports I^2^ = 98% [26]—and our cross-sectional result is as compatible with residual confounding as with a compensatory mechanism. We wish to be explicit about the limits of inference here. A compensatory-then-exhaustion mechanism is a hypothesis that this dataset cannot test, for three reasons. First, the design is cross-sectional: the three groups are different people measured once, not the same people measured repeatedly; so, a difference between groups cannot establish a within-person trajectory, and the findings are described as differences between groups rather than as change over time. Second, neither diabetes duration nor medication was recorded; so, a duration-dependent difference cannot be distinguished from a treatment-associated one; insulin and incretin-based therapies in particular are known to alter metabolic peptide concentrations, and Group B—by virtue of higher HbA1c—is likely to be enriched for both longer disease duration and more intensive therapy. Third, BMI was unavailable, and nesfatin-1 is adiposity-dependent; so, the possibility that Group A differs from the other two groups in adiposity rather than in glycemic stage cannot be excluded. A further caution specific to this cohort is that sex was a stronger predictor of nesfatin-1 than glycemic category; although the group effect survived adjustment and was reproduced within each sex separately, this underscores how readily an unadjusted adipokine comparison can be dominated by a demographic variable. Taken together, we interpret the nesfatin-1 result as a reproducible non-monotonic association between an HbA1c-defined category and a circulating peptide, plausibly but not demonstrably reflecting a stage-dependent compensatory response, and as a hypothesis that warrants prospective testing in cohorts with BMI, disease duration, and medication recorded [18,19].

### 4.3. Vaspin

Vaspin did not differ across glycemic categories on any test applied, including the Kruskal–Wallis omnibus test on untransformed data (*p* = 0.364), one-way ANOVA after log transformation (*p* = 0.221), and the Kruskal–Wallis test after removal of extreme values (*p* = 0.517); nor did it correlate with continuous HbA1c. What distinguished vaspin was its dispersion: the coefficient of variation reached 148% in the control group, the standard deviation exceeded the mean in two of three groups, and the highest single value (6145 ng/mL) was approximately seventeen times the control-group median. This is a distributional feature rather than a between-group signal, and it is the reason a mean-based omnibus test on the raw data returns a nominally significant result (Welch’s ANOVA *p* = 0.015) that does not survive any robust reanalysis. We regard the rank-based and log-scale results as the valid ones and report the parametric result only to document the discrepancy. Such heterogeneity is consistent with prior reports that vaspin tracks adiposity more closely than glycemic control per se [29,30], and studies demonstrating elevated vaspin in obesity and T2DM have generally done so after adjustment for or stratification by BMI [27,28,33]—which was not possible here and is, for vaspin specifically, the most damaging of our data gaps. The wide dispersion observed here is reported as a finding of the present study. Recent evidence has also shifted the context for this null result: in more than 17 000 participants from the PURE and ORIGIN cohorts, higher vaspin predicted incident T2DM (16% per SD) and was linked by Mendelian randomisation to reduced gluteofemoral adiposity [31], and vaspin appears causally related to atherogenic lipids [32]. A cross-sectional comparison of prevalent glycemic categories such as ours is poorly suited to detect a marker whose signal is prospective and adiposity-distribution-dependent, and our finding should be read in that light rather than as contradicting those data. Notably, no meta-analysis of circulating vaspin concentrations in type 2 diabetes has appeared since 2014 [30]; so, the cross-sectional evidence base for this analyte remains thin.

### 4.4. Interpretation of the Secondary Biochemical Parameters

The clinical and biochemical parameters in Table 2 were tested formally rather than described numerically. Of the nine parameters, only HbA1c, fasting glucose, triglycerides, and eGFR differed significantly among groups. The triglyceride elevation in both diabetic groups relative to controls is expected in T2DM and consistent with insulin-resistant dyslipidemia, and triglycerides also correlated with HbA1c across the cohort (ρ = 0.295, *p* < 0.001). The eGFR gradient is, as shown in Section 3.2, an age artefact rather than a renal-function gradient tracking glycemic burden. Total cholesterol, HDL-C, LDL-C, ALT, and AST did not differ; so, the numerically higher values in Group B for several of these parameters should not be read as evidence of more advanced metabolic dysregulation. None of these parameters was used as a covariate in the primary models, which were prespecified to adjust for age and sex only; treating them as mediators or confounders would require a larger sample and an explicit causal model.

### 4.5. Limitations

The limitations of this study are substantial and, in our judgement, determine how far its findings can be taken. First and most important, body mass index was not recorded, and no surrogate measure of adiposity was retrievable. All three adipokines studied are strongly adiposity-dependent, and it is therefore not possible to determine whether the nesfatin-1 difference reflects glycemic category per se or unmeasured differences in adiposity between Group A and the other two groups. This is the single largest gap relative to comparable published work; it is the reason we have tempered the claims in the Abstract and Conclusions from an attribution to glycemic stage to a description of a difference between HbA1c-defined categories, and it must be addressed in any follow-up study. Second, diabetes duration and current antidiabetic medication were unavailable, which is more than a minor methodological gap: it means the observed difference between Group A and Group B may reflect treatment exposure or disease duration rather than glycemia, and this is precisely the distinction the compensatory hypothesis discussed in Section 4.2 would require. Third, the cross-sectional design precludes causal inference and cannot support trajectory language of any kind. Fourth, the control group was defined by HbA1c <6.0% without an accompanying fasting-glucose criterion, and 23 of 54 controls (42.6%) proved to have impaired fasting glucose, while only 22 of 54 (40.7%) met neither prediabetes criterion; although the primary results were unchanged when the control group was restricted to those with fasting glucose < 100 mg/dL, the comparator group is best described as non-diabetic rather than euglycemic, and a metabolically cleaner control group would strengthen any replication. We also did not systematically exclude controls with obesity, cardiovascular, renal, or inflammatory disease, or medications affecting metabolism. Fifth, the three groups were not matched on age, and the control group was 13–17 years younger than the diabetic groups; although age was not independently associated with any adipokine and all group comparisons are reported with age adjustment, residual confounding by age-related factors cannot be excluded, and the age difference fully accounts for the eGFR pattern. Sixth, the sample was predominantly female (73.3%), and sex proved to be a stronger determinant of nesfatin-1 than glycemic category; the group effect was reproduced within each sex, but the small number of men (n = 43) limits the precision of sex-stratified estimates and the female predominance limits generalisability. Seventh, total rather than acylated ghrelin was measured, and single fasting measurements of peptides with pulsatile or prandially modulated secretion may misrepresent habitual concentrations. Eighth, glycemic status was analysed both categorically and continuously, but a multivariable model adjusted for BMI—the analysis the literature most requires—could not be fitted. Ninth, no a priori power calculation was performed; with 52–55 participants per group, this study had adequate power for moderate-to-large effects (the significant nesfatin-1 contrasts corresponded to Hedges’ g of 0.57 and 0.83) but was underpowered for the small effects that would be expected for ghrelin and vaspin; so, those null findings should be read as “no moderate or large difference detected” rather than as evidence of equivalence. Tenth, a proportion of measured values fell outside the calibrated standard-curve range of the corresponding assay, and so those concentrations were obtained by extrapolation beyond the highest standard (42 of 161 ghrelin values, 23 nesfatin-1 values and 21 vaspin values); this adds uncertainty to the absolute concentrations reported here, although it does not affect the rank ordering on which the primary analyses depend. Eleventh, 17 of the 55 participants in Group A had an HbA1c of 6.0–6.4%, below the diagnostic threshold for diabetes; all had a previously established, physician-documented diagnosis and were receiving care for type 2 diabetes, but Group A is consequently weighted towards the better-controlled end of its range. Finally, this was a single-center study conducted in one region of Türkiye, and the findings require external replication.

## 5. Conclusions

In this cross-sectional comparative study, circulating nesfatin-1—but not ghrelin or vaspin—differed significantly between HbA1c-defined glycemic-control categories, and did so non-monotonically, with the highest concentrations in participants with intermediate glycemic control (HbA1c 6.0–8.4%) and no difference between those with poor control (HbA1c ≥ 8.5%) and non-diabetic controls. The difference was of moderate to large magnitude, survived adjustment for age and sex, and was reproduced in both sexes and after restriction of the control group to participants with normal fasting glucose. Ghrelin and vaspin did not differ across categories, and vaspin was characterised chiefly by pronounced inter-individual heterogeneity. Because body mass index, diabetes duration, and antidiabetic medication were not available, these differences cannot be attributed to glycemic status independently of adiposity or treatment; and because the design is cross-sectional, they describe differences between groups of individuals and not a change over time within individuals. The findings are therefore exploratory and hypothesis-generating. They should be confirmed in larger, prospective, BMI- and medication-adjusted cohorts, using continuous glycemic measures and ideally repeated sampling, before nesfatin-1, ghrelin, or vaspin can be considered clinically useful biomarkers of glycemic stage in type 2 diabetes.

## Figures and Tables

**Table 1:** Demographic characteristics of the study groups. Values are n (%), mean ± SD, or median (IQR).

Characteristic	Group A (HbA1c 6.0–8.4%) (n = 55)	Group B (HbA1c ≥ 8.5%) (n = 52)	Control (HbA1c < 6.0%) (n = 54)	*p*-Value
Female, n (%)	41 (74.5)	37 (71.2)	40 (74.1)	0.913 a
Male, n (%)	14 (25.5)	15 (28.8)	14 (25.9)	—
Age, years, mean ± SD	60.5 ± 11.1	63.9 ± 11.4	47.2 ± 12.7	<0.001 b
Age, years, median (IQR)	58.0 (52.5–68.5)	62.5 (56.0–72.0)	48.0 (40.5–54.8)	—
Age range, years	39–92	46–92	20–79	—

IQR: interquartile range. a—Chi-square test (χ^2^ = 0.182, df = 2). b—One-way ANOVA (F(2,158) = 30.27, η^2^ = 0.277); Tukey post hoc: Group A versus control *p* < 0.001, Group B versus control *p* < 0.001, Group A versus Group B *p* = 0.289. Age was normally distributed in all three groups (Shapiro–Wilk *p* ≥ 0.068) with homogeneous variances (Levene’s *p* = 0.900).

**Table 2:** Clinical and biochemical characteristics of the study groups with omnibus and post hoc between-group comparisons.

Parameter	Group A (n = 55)	Group B (n = 52)	Control (n = 54)	Omnibus *p*-Value	Post Hoc (Adjusted *p*)
HbA1c (%)	6.91 ± 0.66 6.90 (6.40–7.25)	10.25 ± 1.50 9.80 (9.10–11.00)	5.44 ± 0.30 5.50 (5.20–5.70)	<0.001	A vs. C < 0.001; B vs. C < 0.001; A vs. B < 0.001
Fasting glucose (mg/dL)	140.7 ± 43.8 129 (114–151)	239.5 ± 77.8 234 (181–292)	100.4 ± 11.9 99 (92–108)	<0.001	A vs. C < 0.001; B vs. C < 0.001; A vs. B < 0.001
eGFR (mL/min/1.73 m^2^)	82.4 ± 16.7 84 (71–96)	87.2 ± 19.4 88 (81–97)	96.8 ± 15.0 98 (88–105)	<0.001	A vs. C < 0.001; B vs. C 0.014; A vs. B 0.592
Total cholesterol (mg/dL)	197.4 ± 50.7 192 (155–240)	206.7 ± 48.1 197 (174–235)	208.7 ± 38.0 209 (184–231)	0.394	not performed
Triglycerides (mg/dL)	177.3 ± 87.6 158 (103–208)	206.9 ± 150.3 166 (128–213)	138.6 ± 72.6 122 (87–162)	0.001	A vs. C 0.015; B vs. C 0.001; A vs. B 1.000
HDL-C (mg/dL)	53.4 ± 9.3 53 (47–58)	53.3 ± 8.6 53 (49–58)	57.2 ± 11.9 55 (47–64)	0.269	not performed
LDL-C (mg/dL)	119.6 ± 65.0 108 (81–133)	152.8 ± 100.6 123 (98–154)	123.9 ± 49.4 115 (99–135)	0.113	not performed
ALT (U/L)	20.1 ± 9.5 17 (14–24)	23.6 ± 18.8 18 (15–25)	19.4 ± 7.6 17 (14–25)	0.670	not performed
AST (U/L)	19.1 ± 6.2 18 (16–22)	19.7 ± 9.3 17 (14–23)	18.7 ± 3.9 18 (16–21)	0.659	not performed

eGFR: estimated glomerular filtration rate; ALT: alanine aminotransferase; AST: aspartate aminotransferase. Omnibus *p*-values are from the Kruskal–Wallis test except for total cholesterol, for which one-way ANOVA was used (normally distributed, homogeneous variances). Post hoc *p*-values are Dunn’s test with Bonferroni adjustment (Tukey’s HSD for total cholesterol). For non-normally distributed variables, the median (IQR) is the primary summary; mean ± SD is given for comparability with the published literature.

**Table 3:** Circulating ghrelin, nesfatin-1, and vaspin concentrations by glycemic group (mean ± SD and median [IQR]).

Adipokine	Group A (n = 55)	Group B (n = 52)	Control (n = 54)
Ghrelin (ng/mL)			
mean ± SD	8.17 ± 3.09	8.04 ± 2.07	8.44 ± 2.40
median (IQR)	9.35 (6.69–10.03)	7.61 (6.63–9.68)	7.82 (6.47–10.84)
range	0.02–12.69	3.35–12.52	4.67–12.52
CV (%)	37.8	25.8	28.4
Nesfatin-1 (pg/mL)			
mean ± SD	792.4 ± 250.2	545.0 ± 340.0	622.5 ± 338.4
median (IQR)	824.2 (631.7–959.4)	599.7 (259.9–808.1)	650.1 (443.0–847.5)
range	19.8–1215.0	2.5–1144.3	5.9–1286.8
CV (%)	31.6	62.4	54.4
Vaspin (ng/mL)			
mean ± SD	400.1 ± 279.6	693.9 ± 755.5	616.8 ± 914.8
median (IQR)	331.1 (183.8–511.6)	404.5 (195.9–835.9)	360.8 (218.1–649.1)
range	67.9–1323.7	72.0–3248.4	25.7–6145.4
CV (%)	69.9	108.9	148.3
geometric mean	318.8	428.6	376.1

**Table 4:** Omnibus and post hoc between-group comparisons for ghrelin, nesfatin-1, and vaspin, with effect sizes and 95% confidence intervals.

Adipokine/Comparison	Test Statistic	*p*-Value	Effect Size (95% CI)
Ghrelin—omnibus (KW)	H(2) = 0.641	0.726	ε^2^ = 0.004
Group A vs. Control	z = 0.220	0.826 (adj. 1.000)	r = 0.02; HL 0.04 (−0.97 to 1.02) ng/mL
Group B vs. Control	z = −0.559	0.576 (adj. 1.000)	r = −0.05; HL −0.22 (−1.24 to 0.57) ng/mL
Group A vs. Group B	z = 0.779	0.436 (adj. 1.000)	r = 0.10; HL 0.41 (−0.45 to 1.57) ng/mL
Nesfatin-1—omnibus (KW)	H(2) = 15.163	<0.001	ε^2^ = 0.095
Group A vs. Control	z = 2.745	0.006 (adj. 0.018)	r = 0.31; HL 155.2 (45.2 to 265.8) pg/mL; g = 0.57 (0.19–0.95)
Group B vs. Control	z = −1.030	0.303 (adj. 0.909)	r = −0.12; HL −67.5 (−224.2 to 52.0) pg/mL; g = −0.23 (−0.61–0.15)
Group A vs. Group B	z = 3.753	<0.001 (adj. <0.001)	r = 0.42; HL 247.7 (120.8 to 380.5) pg/mL; g = 0.83 (0.43–1.22)
Vaspin—omnibus (KW)	H(2) = 2.019	0.364	ε^2^ = 0.013
Group A vs. Control	z = −0.796	0.426 (adj. 1.000)	r = −0.08; HL −34.5 (−130.0 to 55.7) ng/mL
Group B vs. Control	z = 0.625	0.532 (adj. 1.000)	r = 0.06; HL 33.2 (−72.2 to 171.6) ng/mL
Group A vs. Group B	z = −1.416	0.157 (adj. 0.470)	r = −0.17; HL −72.6 (−216.5 to 24.4) ng/mL
Nesfatin-1—sensitivity analyses			
controls with FPG < 100 mg/dL only (n = 29)	—	<0.001 (KW)	—
women only (n = 118)	H(2) = 10.646	0.005	—
men only (n = 43)	H(2) = 11.392	0.003	—
van der Waerden rank ANCOVA (age, sex)	F = 8.494	<0.001	—

KW: Kruskal–Wallis test; HL: Hodges–Lehmann median difference (Group listed first minus Group listed second) with distribution-free 95% confidence interval; r: rank-biserial correlation; g: Hedges’ g with 95% confidence interval; ε^2^: epsilon-squared, computed as H/(N − 1). Post hoc *p*-values are Dunn’s test with Bonferroni adjustment for three comparisons within each analyte; post hoc testing was performed only where the omnibus test was significant, and is shown for completeness elsewhere. Sensitivity analyses are reported in full in Table S3.

**Table 5:** Adjusted and continuous analyses: Spearman correlations with HbA1c, and ANCOVA of each adipokine on glycemic group with adjustment for age and sex.

Analysis	Ghrelin	Nesfatin-1	Vaspin
Spearman ρ with HbA1c, whole cohort (n = 161)	0.007 (*p* = 0.925)	−0.084 (*p* = 0.290)	0.067 (*p* = 0.401)
Spearman ρ with HbA1c, diabetic participants only (n = 107)	−0.044 (*p* = 0.654)	−0.373 (*p* < 0.001)	0.164 (*p* = 0.092)
Jonckheere–Terpstra ordered trend (C → A → B)	z = −0.588 (*p* = 0.556)	z = −0.956 (*p* = 0.339)	z = 0.632 (*p* = 0.528)
Linear HbA1c β per 1% (adjusted for age, sex)	−0.01 (*p* = 0.953)	−37.2 (−58.7 to −15.7; *p* < 0.001)	18.6 (*p* = 0.507)
Quadratic HbA1c^2^ β (nested F test)	−0.01 (*p* = 0.818)	−8.32 (*p* = 0.065)	−13.2 (*p* = 0.255)
ANCOVA group effect, adjusted for age and sex	F = 0.188 (*p* = 0.829)	F = 10.962 (*p* < 0.001)	F = 2.601 (*p* = 0.077)
Group A vs. Control, adjusted β	−0.25 (−1.31 to 0.80); *p* = 0.636	141.7 (29.3 to 254.1); *p* = 0.014	−195.3 (−488.9 to 98.2); *p* = 0.191
Group B vs. Control, adjusted β	−0.34 (−1.46 to 0.78); *p* = 0.552	−100.4 (−219.8 to 19.0); *p* = 0.099	109.5 (−202.3 to 421.3); *p* = 0.489
ANCOVA age effect	F = 0.012 (*p* = 0.912)	F = 1.200 (*p* = 0.275)	F = 0.122 (*p* = 0.728)
ANCOVA sex effect (male vs. female)	F = 6.269 (*p* = 0.013)	F = 56.134 (*p* < 0.001)	F = 1.561 (*p* = 0.213)
male vs. female, adjusted β	−1.13 (−2.02 to −0.24)	−359.6 (−454.4 to −264.8)	−156.6 (−404.2 to 91.0)

β coefficients are unstandardised and expressed in the units of the adipokine (ng/mL for ghrelin and vaspin, pg/mL for nesfatin-1); 95% confidence intervals are given in parentheses where the coefficient is the quantity of interest. ANCOVA models are of the form adipokine ~ glycemic group + age + sex; the group effect is a Type II F test on 2 degrees of freedom with the control group as reference. The quadratic term was tested by a nested F test comparing the model with and without HbA1c^2^. C: control group; A: Group A; B: Group B.

## Data Availability

The data presented in this study are available on request from the corresponding author. The full de-identified participant-level dataset and the analysis scripts used for the revised statistical analysis are available from the corresponding author on reasonable request.

## Supplementary Materials

The following supporting information can be downloaded at: https://www.mdpi.com/article/10.3390/biomedicines14092055/s1. Table S1, Distributional assessment of every continuous variable, by group; Table S2, The pairwise comparisons reported in the previous version of the manuscript, with the corresponding results from the revised omnibus-and-post-hoc analysis; Table S3, Full sensitivity-analysis results; Table S4, Bootstrap confidence intervals for median differences.
