# Supplementary material for: Circulating Ghrelin, Nesfatin-1, and Vaspin Levels Across Glycemic Control Categories in Type 2 Diabetes Mellitus: A Cross-Sectional Study
Source: Biomedicines. 2026 Sep 12;14(9):2055. doi: 10.3390/biomedicines14092055 (PMC13604063; doi:10.3390/biomedicines14092055)
Supplement: Supplementary file 1 [file biomedicines-14-02055-s001.zip › biomedicines-4557052-supplementary.pdf]

Supplementary Materials

Table S1. Distributional assessment of every continuous variable, by group.

Variable	Group	n	Skewness	Kurtosis	Shapiro–Wilk p	Normality (S–W)	Verdict applied
Age (years)	Group A	55	0.57	-0.05	0.152	normal	normal
	Group B	52	0.47	-0.49	0.068	normal	
	Control	54	-0.04	0.10	0.625	normal	
HbA1c (%)	Group A	55	0.62	-0.36	0.007	non-normal	non-normal
	Group B	52	0.86	-0.27	<0.001	non-normal	
	Control	54	-0.62	-0.56	0.005	non-normal	
Fasting glucose (mg/dL)	Group A	55	1.68	2.82	<0.001	non-normal	non-normal
	Group B	52	0.39	-0.25	0.482	normal	
	Control	54	1.59	4.06	<0.001	non-normal	
eGFR (mL/min/1.73 m²)	Group A	55	-0.36	-0.48	0.072	normal	non-normal
	Group B	52	-0.62	1.86	0.029	non-normal	
	Control	54	-0.01	-0.19	0.905	normal	
Total cholesterol (mg/dL)	Group A	55	0.26	-0.61	0.282	normal	normal
	Group B	52	0.62	0.67	0.201	normal	
	Control	54	0.25	0.09	0.920	normal	
Triglycerides (mg/dL)	Group A	55	1.11	1.02	<0.001	non-normal	non-normal
	Group B	52	2.66	7.33	<0.001	non-normal	
	Control	54	2.05	5.38	<0.001	non-normal	
HDL-C (mg/dL)	Group A	55	0.44	0.01	0.184	normal	non-normal
	Group B	52	0.01	-0.47	0.435	normal	
	Control	54	0.88	0.42	0.004	non-normal	
LDL-C (mg/dL)	Group A	55	2.95	10.30	<0.001	non-normal	non-normal
	Group B	52	1.76	1.81	<0.001	non-normal	
	Control	54	3.28	16.23	<0.001	non-normal	
ALT (U/L)	Group A	55	1.48	2.81	<0.001	non-normal	non-normal
	Group B	52	3.94	19.52	<0.001	non-normal	
	Control	54	1.31	1.22	<0.001	non-normal	
AST (U/L)	Group A	55	1.56	2.99	<0.001	non-normal	non-normal
	Group B	52	2.70	10.25	<0.001	non-normal	
	Control	54	0.64	0.38	0.101	normal	
Ghrelin (ng/mL)	Group A	55	-1.01	0.57	<0.001	non-normal	non-normal
	Group B	52	0.06	-0.43	0.361	normal	
	Control	54	0.19	-1.43	<0.001	non-normal	
Nesfatin-1 (pg/mL)	Group A	55	-0.87	1.08	0.019	non-normal	non-normal
	Group B	52	-0.06	-1.17	0.023	non-normal	
	Control	54	-0.35	-0.59	0.016	non-normal	
Vaspin (ng/mL)	Group A	55	1.38	1.74	<0.001	non-normal	non-normal
	Group B	52	1.84	2.70	<0.001	non-normal	

	Control	54	4.48	23.28	<0.001	non-normal

A variable was treated as non-normally distributed if the Shapiro–Wilk test rejected normality ($p \le 0.05$) in at least one group. Kurtosis is excess kurtosis (normal = 0). Note that nesfatin-1 passed the Kolmogorov–Smirnov (Lilliefors) test in all three groups ($p = 0.381, 0.097, 0.106$) but fails Shapiro–Wilk; the non-parametric analysis was therefore adopted, and both parametric and rank-based results are reported in the main text so that the reader can verify that the conclusion is unaffected.

Table S2. The pairwise comparisons reported in the previous version of the manuscript, with the corresponding results from the revised omnibus-and-post-hoc analysis.

Adipokine	Comparison	Previous version: test and statistic	Previous p-value	Previous verdict (Bonferroni α = 0.0056)	Revised analysis: Dunn z	Revised p (Bonferroni-adjusted)	Revised verdict (α = 0.05)
Ghrelin	A vs Control	Mann–Whitney z = −0.142	0.887	not significant	0.220	1.000	not significant
	B vs Control	Mann–Whitney z = −0.477	0.663	not significant	−0.559	1.000	not significant
	A vs B	Mann–Whitney z = −0.857	0.391	not significant	0.779	1.000	not significant
Nesfatin-1	A vs Control	Welch t = 2.977	0.004	significant	2.745	0.018	significant
	B vs Control	t-test t = −1.176	0.242	not significant	−1.030	0.909	not significant
	A vs B	Welch t = 4.268	<0.001	significant	3.753	<0.001	significant
Vaspin	A vs Control	Mann–Whitney z = −0.739	0.460	not significant	−0.796	1.000	not significant
	B vs Control	Mann–Whitney z = −0.566	0.572	not significant	0.625	1.000	not significant
	A vs B	Mann–Whitney z = −0.739 a	0.460 a	not significant	−1.416	0.470	not significant

a The submitted version reported z = −0.739, p = 0.460 for the vaspin A versus B comparison, identical to the A versus control row above it. Re-analysis of the raw data gives z = −1.471, p = 0.142 on the same Mann–Whitney test, indicating that the submitted entry was a transcription duplication of the row above; the corresponding Dunn statistic in the revised framework is z = −1.416, unadjusted p = 0.157. The verdict is unaffected. Sign conventions differ between the two analyses (the previous version reported the SPSS Mann–Whitney z, the revised analysis reports the Dunn z of the first group minus the second), so the magnitudes rather than the signs should be compared. No verdict changes between the two frameworks: the revised analysis reproduces every conclusion of the original while using a conventional procedure and a conventional significance threshold.

Table S3. Full sensitivity-analysis results.

Each analysis repeats the primary three-group comparison under a different set of assumptions or on a different subset of the data. The nesfatin-1 result is significant in every analysis; the ghrelin and vaspin results are non-significant in every analysis.

Analysis	Assumption relaxed or subset used	Ghrelin	Nesfatin-1	Vaspin
Primary: Kruskal–Wallis, all participants	none (primary analysis)	H = 0.641, p = 0.726	H = 15.163, p < 0.001	H = 2.019, p = 0.364
One-way ANOVA, untransformed	assumes normality and equal variances	F = 0.344, p = 0.709	F = 8.881, p < 0.001	F = 2.546, p = 0.082
Welch's ANOVA, untransformed b	assumes normality, allows unequal variances	F = 0.432, p = 0.650	F = 10.331, p < 0.001	F = 4.445, p = 0.015 a
Kruskal–Wallis after removal of values meeting Tukey's extreme-outlier criterion (>3 × IQR beyond Q1 or Q3)	removes 5 extreme values (Group B n = 2, control n = 3)	—	—	p = 0.517
One-way ANOVA on natural-log scale	normalises right-skewed data (Shapiro–Wilk p ≥ 0.309 for vaspin)	—	—	F = 1.524, p = 0.221
Kruskal–Wallis with controls restricted to fasting glucose <100 mg/dL (n = 29)	removes controls with impaired fasting glucose	p = 0.529	p = <0.001	p = 0.372
Group A vs Control within that subset	as above	p = 0.749	p = 0.030	p = 0.714
Group B vs Control within that subset	as above	p = 0.308	p = 0.436	p = 0.561
Kruskal–Wallis, women only (n = 118)	removes any influence of the sex imbalance	—	H = 10.646, p = 0.005	—
Kruskal–Wallis, men only (n = 43)	as above	—	H = 11.392, p = 0.003	—
ANCOVA adjusted for age and sex	adjusts for the measured confounders	F = 0.188, p = 0.829	F = 10.962, p < 0.001	F = 2.601, p = 0.077
Rank-based (van der Waerden) ANCOVA, age- and sex-adjusted	adjusts for confounders with no distributional assumption	F = 0.206, p = 0.814	F = 8.494, p = <0.001	F = 1.371, p = 0.257
Female-only ANCOVA adjusted for age (n = 118)	as above, women only	—	F = 5.042, p = 0.008	—
Kruskal–Wallis after excluding the single ghrelin value below the assay's limit of detection (0.016 ng/mL)	removes a below-LOD value	p = 0.634	—	—
Games–Howell post hoc after Welch's ANOVA: A vs Control	parametric post hoc, unequal variances	p = 0.867	p = 0.010	p = 0.226
Games–Howell: B vs Control	as above	p = 0.629	p = 0.470	p = 0.884
Games–Howell: A vs B	as above	p = 0.964	p < 0.001	p = 0.028 a
Jonckheere–Terpstra ordered-trend test (Control → A → B)	tests for a monotonic rather than a categorical difference	z = −0.588, p = 0.556	z = −0.956, p = 0.339	z = 0.632, p = 0.528

a This is the one analysis in which vaspin reaches nominal significance. It assumes normality, which vaspin violates severely (Shapiro–Wilk p < 0.001 in all three groups; coefficient of variation 70–148%; five values more than 3 × IQR above their group median). Every robust reanalysis of the same data — the rank-based primary test, the trimmed test and the analysis on the log scale — returns a non-significant result, so we regard the Welch's ANOVA result as an artefact of a small number of extreme values and report it only for transparency; the Games–Howell post hoc result for the same contrast (p = 0.028) carries the same caveat. b Welch's ANOVA and the Games–Howell procedure were computed for all three analytes as a sensitivity analysis; Levene's test (median-centred) was significant only for nesfatin-1 (p = 0.022), and was non-significant for ghrelin (p = 0.171) and vaspin (p = 0.065). IQR: interquartile range; LOD: limit of detection. Where an analysis is specific to one analyte, the remaining columns are marked "—".

Table S4. Bootstrap confidence intervals for median differences

Adipokine	Comparison	Observed difference in medians	Bootstrap 95% CI (10 000 resamples)	Excludes zero
Ghrelin	A vs Control	1.53 ng/mL	-1.27 to 2.52	no
	B vs Control	-0.22 ng/mL	-2.18 to 1.59	no
	A vs B	1.74 ng/mL	-0.57 to 2.30	no
Nesfatin-1	A vs Control	174.05 pg/mL	29.21 to 271.15	yes
	B vs Control	-50.41 pg/mL	-272.64 to 55.74	no
	A vs B	224.46 pg/mL	104.47 to 425.39	yes
Vaspin	A vs Control	-29.78 ng/mL	-136.31 to 121.10	no
	B vs Control	43.67 ng/mL	-91.15 to 255.91	no
	A vs B	-73.45 ng/mL	-265.10 to 84.56	no

Bias-corrected percentile bootstrap, seed 42. The two nesfatin-1 contrasts that are significant in the Dunn post hoc analysis are the only two whose bootstrap confidence interval for the difference in medians excludes zero, which is a further independent confirmation of the primary result.
